# Continuous Flow Removal of Anionic Dyes in Water by Chitosan-Functionalized Iron Oxide Nanoparticles Incorporated in a Dextran Gel Column

**DOI:** 10.3390/nano9081164

**Published:** 2019-08-14

**Authors:** Sang Yeob Lee, Ha Eun Shim, Jung Eun Yang, Yong Jun Choi, Jongho Jeon

**Affiliations:** 1 Department of Applied Chemistry, School of Applied Chemical Engineering, Kyungpook National University, Daegu 41566, Korea; 2 Department of Chemistry, Kyungpook National University, Daegu 41566, Korea; 3 Department of Advanced Process Technology and Fermentation, World Institute of Kimchi, Gwangju 61755, Korea; 4 School of Environmental Engineering, University of Seoul, Seoul 02504, Korea

**Keywords:** iron oxide nanomaterials, anionic dye, purification, immobilization, nanocomposite, remediation, chromatography

## Abstract

This paper describes a novel chromatographic method for efficient removal of anionic dyes from aqueous solutions. Chitosan-coated Fe_3_O_4_ nanoparticles can easily be immobilized on a dextran gel column. Single elution of Evans Blue (EB) solution to the nanoadsorbent-incorporated columns provides high removal efficiency with a maximum adsorption capacity of 243.9 mg/g. We also investigated the influence of initial concentration and solution pH on the removal efficiency of EB. The electrostatic interaction between the adsorbent surface and negatively charged sulfate groups on EB molecules promotes the efficient adsorption of dyes. The equilibrium data matched well with the Langmuir isotherm model, which indicated monolayer dye adsorption onto the adsorbent surface. To extend the application of the current method, we performed further adsorption experiments using other anionic dyes of different colors (Cy5.5, Acid Yellow 25, Acid Green 25, and Acid Red 1). All of these molecules can efficiently be captured under continuous flow conditions, with higher removal efficiency obtained with more negatively charged dyes. These findings clearly demonstrate that the present approach is a useful method for the removal of anionic dye contaminants in aqueous media by adsorption.

## 1. Introduction

With the rapid development of various industries and the growth of manufacturing, the coinciding increase in pollution as a threat to the environment and public health has prompted considerable attention to improve the treatment of wastewater [1,2]. Among a variety of contaminants released into the environment, organic dyes are considered to be one of the most dangerous pollutants; many of these molecules are known to be toxic, mutagenic, and carcinogenic, which can have adverse effects on aquatic creatures and human beings [3,4,5,6,7]. Anionic dyes are particularly troublesome due to their high solubility in water, various harmful effects on living organisms, and large quantities generated from many industrial sources [8,9]. Moreover, these molecules are typically difficult to degrade due to their complex aromatic structures, and thus anionic dyes can persist as contaminants in water for long periods of time. Therefore, various physicochemical and biological processes for dye removal from wastewater have been investigated to solve this urgent environmental issue. Previously reported techniques for alleviation of dye-containing wastewater include adsorption [10,11,12], filtration [13], biodegradation [14], chemical coagulation [15], photocatalysis [16], and electrolysis [17], among other methods. The adsorption technique is regarded as the preferred separation method, and is therefore widely applied to remove organic dyes in aqueous media due to simplicity of design, high efficiency, low cost, and facile and controllable operation. Among various adsorbents, much attention has been paid to the use of metal oxide nanomaterials [18]. In recent years, adsorbents based on Fe_3_O_4_ nanomaterials have been widely investigated for efficient removal of dyes in wastewater due to their low toxicity, easy and inexpensive preparation, and large surface area. To date, most adsorption experiments using Fe_3_O_4_ have been performed using batch processing [19,20,21,22]. The adsorbents are immersed in the wastewater and then additional separation processes are required to remove the dye-adsorbed nanomaterials from the water after adsorption via an external magnetic field.

We previously studied and reported adsorbent-immobilized composite materials to perform continuous flow desalination of radioactive elements in water [23,24,25,26]. These hybrid nanomaterials stabilize the adsorbent and minimize aggregation/agglomeration of the adsorbent during water treatment. Moreover, these techniques allow easier separation of solid adsorbents from the decontaminated water. Our methods provided superior performance, such as rapid and high removal efficiency when compared to known batch processes. Herein, we report a convenient continuous flow process for efficient capture of anionic dyes using Fe_3_O_4_-incorporated dextran gel column. For this study, we have selected chitosan functionalized Fe_3_O_4_ nanoparticles because the cationic nature of chitosan can be helpful for the removal of anionic dyes from aqueous solutions and is also useful for stable immobilization of the nanoadsorbent in the dextran gel beads. Our current method employs affinity chromatography to remove dye contaminants in water, making use of the fact that chitosan-functionalized Fe_3_O_4_ adsorbents on solid supports are known to capture anionic dyes. First, the adsorption performance of the current system is evaluated systemically using Evans Blue (EB) dye and then the same method is further extended to the removal of several other anionic dyes.

## 2. Materials and Methods

### 2.1. Materials

Evans Blue, Acid Red 1, Acid Yellow 25, Acid Green 25, Amberlite^®^-410, Amberlite^®^-900, humic acid (HA), and NaOH were purchased from Sigma-Aldrich (Yongin, Korea). The dye Cyanine 5.5 (Cy5.5) and dextran gel desalting column (PD-10) were supplied by GE Healthcare. Chitosan-coated Fe_3_O_4_ nanomaterials and dextran sulfate-coated Fe_3_O_4_ nanomaterials were purchased from Chemicell (Berlin, Germany). All reagents were of analytical grade and used without further purification. Concentrations of dye solutions were measured using a UV-vis spectrophotometer (Shimadzu, UV-1800, Kyoto, Japan).

### 2.2. Preparation of Chitosan-Fe_3_O_4_-Incorporated Desalting Column (Fe-DC)

A dextran gel desalting column (PD-10) was washed with deionized water (3 × 5 mL) and slowly loaded with a suspension of chitosan-coated Fe_3_O_4_ nanoparticles (2.5 mg) in 10 mL deionized water. After the nanoparticles were incorporated in the cross-linked dextran gels, the column was further washed with pure water to provide Fe-DC. Preparation of Fe-DC could normally be accomplished in 20 min.

### 2.3. Characterization of Adsorbent

The structure of chitosan-Fe_3_O_4_-incorporated dextran gel microbeads was observed using field emission scanning electron microscopy (SEM; FEI Verios 460L, Hillsboro, OR, USA) under high-performance conditions with accelerating voltages up to 15 kV. The elemental composition of the microbeads was analyzed by SEM-energy dispersive X-ray spectroscopy (EDX, AMITEC) analysis with accelerating voltages of up to 20 kV. EDX spectra were recorded in the area scan mode by focusing the electron beam onto a region of the sample surface.

### 2.4. Removal of Anionic Dyes in Water Using Fe-DC

To measure the adsorption capacity of Fe-DC for Evans Blue (EB), an aqueous solution (75 mL) containing 2.5 to 100 μM EB was poured into to Fe-DC and the flow rate was maintained at ~1.5 mL/min. The eluate was collected every 3 mL (×25), and the concentration of each eluate was analyzed by UV-vis absorbance spectroscopy. The adsorption isotherm was analyzed by Langmuir, Freundlich, and Temkin equations as follows.

(1) $$\mathrm{Langmuir}\;\mathrm{equation}:\;\frac{C_{e}}{q_{e}}=\frac{C_{e}}{q_{m}}+\frac{1}{q_{m}K_{L}}$$

(2) $$\mathrm{Freundlich}\;\mathrm{equation}:\;\mathrm{In}q_{e}={\mathrm{InK}}_{F}+\frac{1}{n}\ln C_{e}$$

(3) $$\mathrm{Temkin}\;\mathrm{equation}:\;q_{e}={\mathrm{BInA}}_{t}+\mathrm{BIn}C_{e}$$

where *C*_0_ and *C*_e_ (μmol) are the initial concentration of the dye solution and the residual concentration in the eluate, respectively; *q*_e_ (mg/g) is the amount of dye absorbed per unit mass of absorbent; and *q*_m_ (mg/g) is the maximum adsorption capacity of the adsorbent. K_L_ and K_F_ are the Langmuir adsorption constant and Freundlich constant, respectively. B is the constant related to the heat of sorption (J/mol) at 298 K and A*_t_* is the Temkin isotherm equilibrium binding constant (L/g).

To evaluate removal efficiency of Fe-DC for anionic dye molecules, each solution (10 mL) was added to Fe-DC with ~1.5 mL/min flow rate. The removal efficiency (%) is defined by the following equation to assess the adsorption capability of Fe-DC towards anionic dyes.

(4) $$\mathrm{Removal}\;\mathrm{efficiency}\;\left(\%\right)=\frac{C_{0}-C_{e}}{C_{0}}\times 100$$

## 3. Results and Discussion

### 3.1. Preparation of Chitosan-Fe_3_O_4_-Incorporated Desalting Column (Fe-DC)

**Figure 1:**
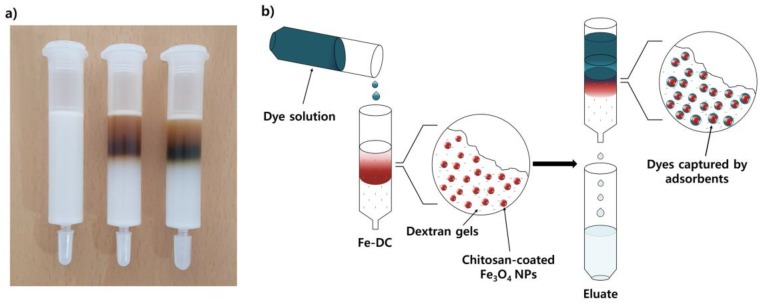
(**a**) Image of desalting columns: (left) bare PD-10, (middle) Fe-DC, and (right) Fe-DC after adsorption of EB; (**b**) schematic illustration of the dye adsorption process from aqueous solutions using Fe-DC under continuous flow conditions (~1.5 mL/min).

The key strategy of this study is illustrated in Figure 1a, whereby the column needs to be prepared before the capture of dye molecules can be performed. The first step is the preparation of the chitosan-Fe_3_O_4_-incorporated desalting column (Fe-DC). To immobilize the adsorbents, chitosan-coated iron oxide nanoparticles (chitosan-Fe_3_O_4_) were added to a commercially available PD-10 desalting column (8.3 mL bed volume). A chitosan-Fe_3_O_4_ (65 nm average hydrodynamic radius) (Figures S1 and S2) suspension (10 mL) was slowly added to the column (Figure S3). The positively charged chitosan-Fe_3_O_4_ nanoparticles (zeta potential = +21.2 at neutral pH) became incorporated by interactions on the surface of negatively charged cross-linked dextran gels.

**Figure 2:**
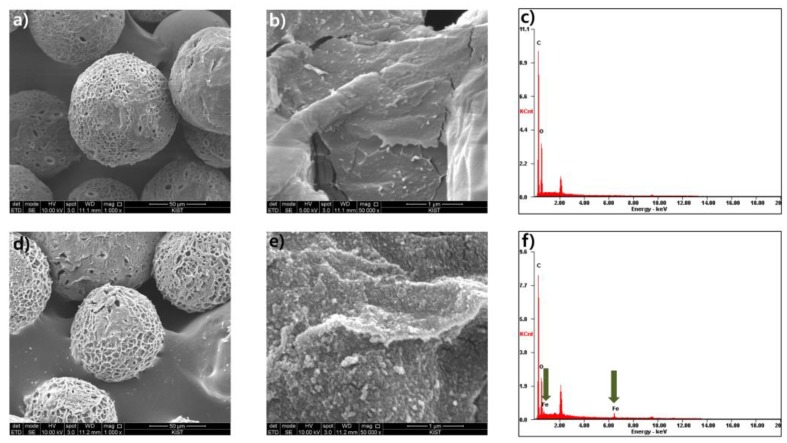
SEM images of (**a**,**b**) dextran gel and (**d**,**e**) chitosan-Fe_3_O_4_-incorporated dextran gel. EDX analysis of (**c**) dextran gel and (**f**) chitosan-Fe_3_O_4_-incorporated dextran gel; arrows indicate the presence of iron.

After chitosan-Fe_3_O_4_ nanoparticles enter the packed bed completely, the resulting column was washed with pure water to provide a chitosan-Fe_3_O_4_-incorporated desalting column (Fe-DC) (Figure 1b). The chitosan-Fe_3_O_4_ nanoparticles incorporated in the desalting column were stable and remain attached, as they do not elute nor undergo aggregation upon continual washing with water or aqueous solutions of 0.1 M NaCl or 0.1 M NaOH. Under acidic conditions, however, both chitosan-Fe_3_O_4_ and the dextran gel are positively charged. Thus, some chitosan-Fe_3_O_4_ nanoparticles begin to elute from the column when the pH of eluent is strongly acidic (pH < 3). To further investigate whether the incorporation of the adsorbent is caused by the electrostatic interaction between chitosan-Fe_3_O_4_ nanoparticles and the dextran gel beads, negatively charged dextran sulfate–coated Fe_3_O_4_ nanoparticles were added at neutral pH. As expected, the nanoparticles were not immobilized by the dextran gels and were quickly eluted from the PD-10 column. These observations indicate that an electrostatic interaction between two oppositely charged components, namely positively charged chitosan-Fe_3_O_4_ nanoparticles and negatively charged dextran gel, lead to immobilization when pH ≥ 4. A series of magnified SEM images of Fe-DC (Figure 2 and Figure S4) indicate incorporation of chitosan-Fe_3_O_4_ on the surface of the dextran microbeads. In addition, elemental analysis using EDX exhibits peaks for iron from the nanoparticles, and carbon and oxygen from dextran (Figure 2f), clearly demonstrating incorporation of the chitosan-Fe_3_O_4_ nanoparticles as adsorbents on the PD-10 dextran gel desalting column.

### 3.2. Adsorption of Evans Blue (EB) Dye Using Fe-DC

**Figure 3:**
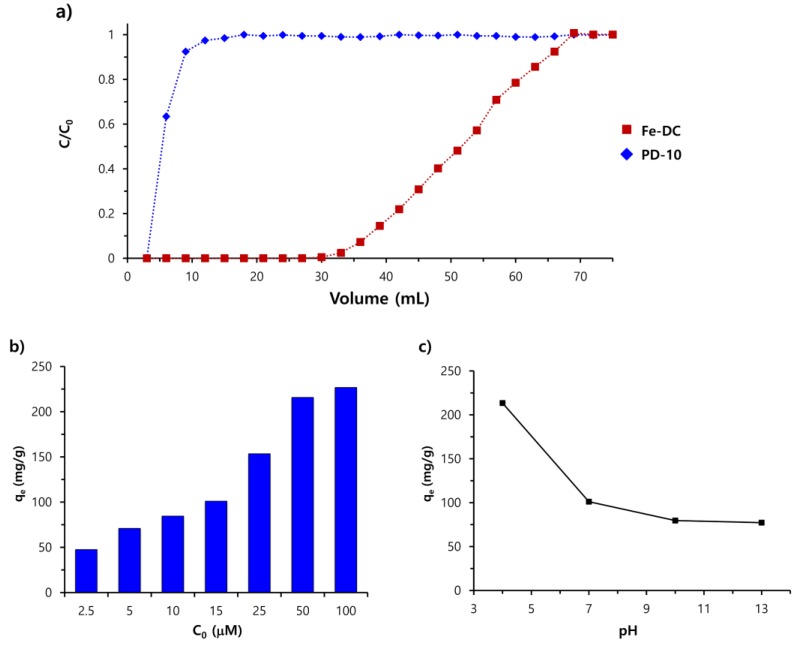
(**a**) Breakthrough curve for EB adsorption as a function of time using the Fe-DC column (where *C*_0_ is 2.5 μM of dye and *C* is the measured concentration in the eluent) and bare PD-10, (**b**) effect of initial dye concentration on the adsorption efficiency, (**c**) effect of the initial solution pH on the adsorption efficiency of the Fe-DC column.

To initially investigate the removal efficiency of an anionic dye from an aqueous solution under continuous flow conditions, an aqueous solution of EB (2.5 μM, 75 mL) was added to Fe-DC (Figure 1b and Figure S5) at a rate of 1.5 mL/min. The concentration of dye in the eluate can be measured with UV-vis absorbance spectroscopy and the removal capability determined by dividing the concentration of the eluate by the initial concentration of the dye (*C*/*C*_0_). Figure 3a shows that EB is efficiently captured by Fe-DC, with >99% removal efficiency maintained until 30 mL of the dye solution is passed through the Fe-DC. As shown in Figure 1a (right), EB is captured on Fe-DC after elution is complete. Dextran gel alone (PD-10 without incorporated chitosan-Fe_3_O_4_ nanoparticles) retains only a small fraction of EB non-specifically, with most of the dye molecules eluted almost immediately (Figure 3a), demonstrating that the removal of EB was mediated by chitosan-Fe_3_O_4_ nanoparticles incorporated in the column.

### 3.3. Adsorption Isotherm Modeling

After confirming that the incorporation of chitosan-Fe_3_O_4_ nanoparticles is indeed necessary for immobilization of EB, the adsorption efficiency and effect of pH were studied. Adsorption efficiency was studied using various concentrations of EB ranging from 2.5 to 100 μM. The adsorption capacity (*q*_e_) of Fe-DC increases with the increasing initial concentration of EB (Figure 3b). The pH of the solution may also affect the adsorption performance by changing the surface properties of chitosan-Fe_3_O_4_ nanoparticles. To investigate the effect of solution pH, aqueous solutions of EB (15 μM) at pH 4, 7, 10, and 13 were added to the Fe-DC. As shown in Figure 3c, the *q*_e_ tends to increase with decreasing pH (down to pH 4), which indicates that EB can be adsorbed more easily under slightly acidic conditions rather than in alkaline media. This phenomenon can be attributed to the fact that as the pH decreases, chitosan-Fe_3_O_4_ becomes protonated (i.e., the amine group of chitosan), and thus the overall surface charge will become more positive, which results in enhanced adsorption efficiency by the stronger electrostatic attraction between the cationic ammonium groups on chitosan-Fe_3_O_4_ and anionic sulfate groups on EB [27]. At a higher pH, more hydroxyl ions (OH^-^) in the aqueous media compete with negatively charged dyes, thus leading to less overall removal efficiency of EB. These mechanistic considerations explain how better adsorption capacity was obtained at a lower pH. To further investigate the effects of other contaminants on adsorption capacity, we prepared a 15 μM EB solution containing humic acid (HA), a heterogeneous macromolecule mainly found in soil and water. The solution was poured into Fe-DC under the abovementioned conditions. As shown in Figure S6, when the amount of HA was twice as much as that of EB, the observed *q*_e_ equaled 70.8 mg/g, which is ~30% less than the value obtained for pure water (101.08 mg/g). This finding was ascribed to the fact that HA functional groups (e.g., catechol units) can also bind to the surface of Fe_3_O_4_ to occupy adsorption sites, which results in reduced adsorption ability.

**Figure 4:**
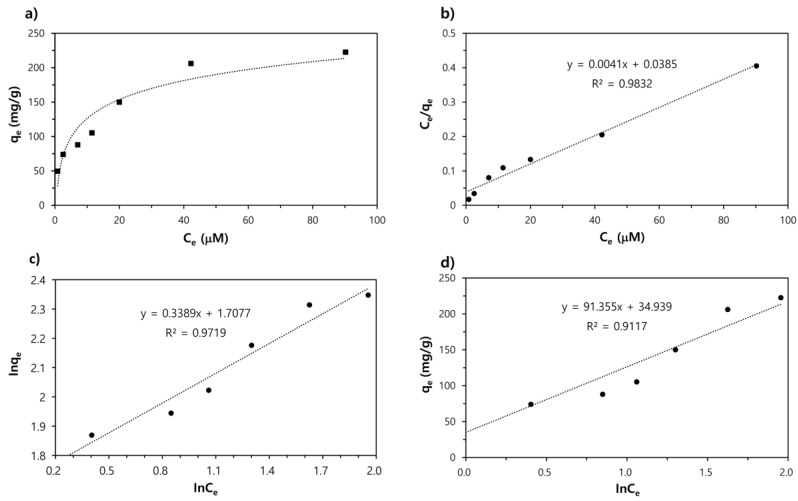
(**a**) Adsorption isotherm of Evans Blue to Fe-DC at pH 7. Isotherm plots and fits of the (**b**) Langmuir, (**c**) Freundlich, and (**d**) Temkin models. Equations used for fitting the data are listed in Section 2.4.

To describe the adsorption isotherm of EB, the adsorption data are analyzed using three isothermal equations (Langmuir, Freundlich, and Temkin models) as described in Section 2.4. The linear fittings of the graphs with Equations (1)–(3) are shown in Figure 4, and the corresponding adsorption parameters for these models are summarized in Table 1. According to the calculated values, the Langmuir model matches more closely than the other two models, as it provides a higher correlation coefficient value (*R*^2^ = 0.9832). This result suggests that the adsorption of EB by the Fe-DC is monolayer adsorption [28]. The obtained maximum adsorption capacity (*q*_max_) for EB is found to be 243.902 mg/g of the adsorbent.

### 3.4. The Removal of Five Anionic Dyes Using Fe-DC

**Figure 5:**
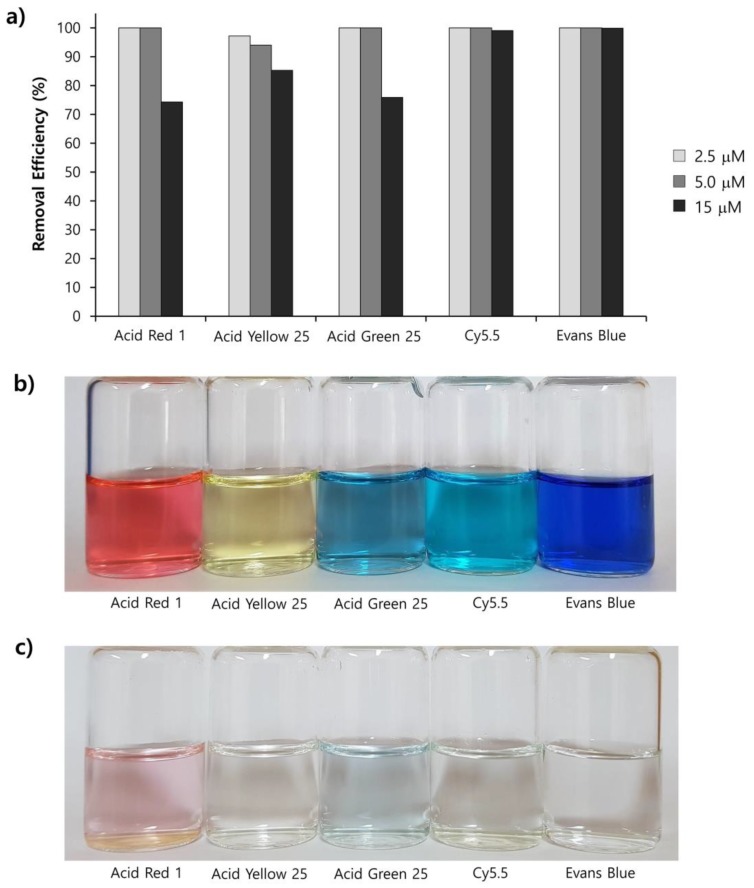
(**a**) Removal efficiencies of anionic dyes using Fe-DC (2.5, 5.0, and 15.0 μM initial concentration) at pH 7. Photographic images (**b**) before and (**c**) after purification of anionic dyes (15.0 μM initial concentration).

To apply the present method to the purification of other anionic molecules, we prepared four additional aqueous dye solutions that display different colors. Table 2 shows the structures and their physical characters of dyes including charges (i.e., number of sulfate groups), and maximum UV-vis absorption wavelengths (*λ*_max_). To investigate removal efficiencies, dye solutions with different initial concentrations (2.5, 5.0, and 15 μM) were passed through the Fe-DC. The removal efficiency (%) obtained for the dyes (Figure 5a) can be calculated using Equation (4). At low concentration, most of the dye molecules were efficiently captured by the Fe-DC. With increasing initial dye concentration, the removal efficiencies for anionic dyes gradually decrease. This trend can be explained by considering that dyes at lower initial concentration are more likely to be immobilized while leaving more vacant sites on the chitosan-Fe_3_O_4_ nanoparticle adsorbent. On the other hand, as initial dye concentration increases, the occupied sites of the adsorbent make it more difficult to adsorb additional anionic dye molecules.

The removal of dye molecules by Fe-DC can also be visualized by photographic images of the solutions. As shown in Figure 5b,c, the intensity of the eluate color (after purification) is significantly less compared to the corresponding solution before purification (15 μM initial concentration). Interestingly, better results are obtained with more negatively charged dyes. For example, both EB and Cy5.5 contain four negatively charged sulfate groups, exhibiting better removal efficiencies compared to the other dyes containing only one or two sulfate groups. Previous studies demonstrate that the electrostatic interaction is the dominant mechanism for adsorption of charged organic dyes on the surface of metal oxide nanomaterials [29]. To stabilize the anionic charge on the dyes, interactions between these negatively charged dyes and the chitosan-Fe_3_O_4_ surface facilitate the adsorption process. More importantly, the sulfate group is known to possess a sufficient affinity for use as an anchoring group on the Fe_3_O_4_ nanoparticles [30,31,32]. Therefore, the sulfate anion(s) of the dye can form surface complexes with the adsorbents, leading to better removal capability with the Fe-DC.

Many reports on the adsorption of anionic dyes in aqueous media using various adsorbents are successfully applied in batch processing for removal of dyes [19,20,21,22,33,34,35,36,37,38,39]. However, these processes frequently require an additional process to separate the solid adsorbents from the water after the purification procedure is finished. Compared to these previous reports, the continuous flow system demonstrated in the present study is simpler and more efficient for removal of anionic dye contaminants in water. Fe-DC can be easily prepared in large quantities and in a short time. Notably, with a single elution of dye solution through the Fe-DC, anionic dyes can efficiently be captured by incorporated chitosan-Fe_3_O_4_ nanoparticle adsorbents, and the observed *q*_max_ values are superior to those obtained in previous studies (Table S1).

Anionic exchange resins also possess adsorption capabilities for the removal of anionic dyes [40,41]. For the corresponding comparison experiments, different amounts of Amberlite^®^-410 and Amberlite^®^-900, which feature quaternary ammonium groups on the microbead surface, were incorporated in the same desalting column (Figure S7). Continuous flow experiments for EB removal in an aqueous solution demonstrated that much larger amount of resins (100 mg of Amberlite^®^-410) were necessary to achieve a high removal efficiency of ~98% (Figure S8). On the other hand, Fe-DC contained only 2.5 mg of chitosan-Fe_3_O_4_ nanoparticles, and the corresponding column exhibited a better removal efficiency. This result shows that compared to large-particle-size adsorbents, nanomaterial-based ones can exhibit better adsorption performance in water. Overall, the above advantages strongly demonstrate that affinity chromatography using Fe-DC is a potentially useful method for efficient anionic dye removal.

## 4. Conclusions

In this work, we successfully developed a new purification method to remove anionic dyes from aqueous solutions. The combination of two materials, chitosan-coated Fe_3_O_4_ nanoparticles and a commercially available cross-linked dextran gel, afforded a useful affinity column, Fe-DC. The three-dimensional structure of immobilized adsorbents was characterized by the SEM-EDX analysis. Fe-DC conveniently separates dye molecules using simple elution of contaminant solutions. Moreover, this procedure does not involve magnetic separation of adsorbents. Factors such as initial dye concentration, initial solution pH, and molecular charge have a noticeable impact on the adsorption capacity and removal efficiency. The experimental results of the Fe-DC adsorption isotherms fit well to the Langmuir model. We have demonstrated adsorption of five anionic dyes using a continuous flow system, with the more negatively charged dyes exhibiting larger maximum theoretical adsorption capacity. The purification capability of the current method compares favorably with most previous results using batch processing. Our results suggest that Fe-DC is a promising adsorbent system worth investigating for large-scale removal of anionic dyes.

## Figures and Tables

**Table 1:** Obtained isotherm model parameters for the adsorption of Evans Blue.

Isotherm Equation	Equation	*q*_m_ (mg/g)	K_L_ (L/mg)	K_F_ (L^−1/n^ mg^1−1/n^)	*n*	A*_t_* (L/g)	B (J/mol)	*R* ^2^
Langmuir model	(1)	243.902	0.106	–	–	–	–	0.9832
Freundlich model	(2)	–	–	5.516	2.95	–	–	0.9719
Temkin model	(3)	–	–	–	–	1.465	91.355	0.9117

**Table 2:** Chemical structures and selected properties of anionic dyes.

Name (MW)	Structure	Charge at Neutral pH (Number of Sulfates)	*λ*_max_
Acid Yellow 25 (549.5518)	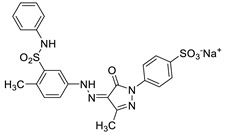	−1 (1)	392 nm
Acid Red 1 (509.4145)	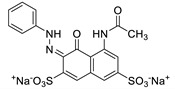	−2 (2)	506, 532 nm
Acid Green 25 (622.5735)	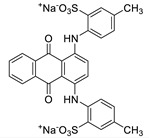	−2 (2)	608, 642 nm
Cy5.5 (1005.9775)	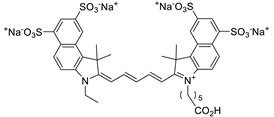	−3 (4)	682 nm
Evans Blue (960.7931)	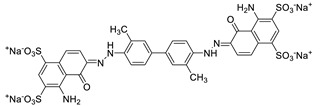	−4 (4)	620 nm

## Supplementary Materials

The following materials are available online at https://www.mdpi.com/2079-4991/9/8/1164/s1, Figure S1: Zeta potential of chitosan-coated Fe_3_O_4_ nanoparticles, Figure S2: Hydrodynamic size of chitosan-coated Fe_3_O_4_ nanoparticles, Figure S3: Incorporation of chitosan-coated Fe_3_O_4_ nanoparticles in the dextran gel column (PD-10), Figure S4: (a,b) SEM images of EB-adsorbed dextran gel beads, (c) EDX analysis of EB-adsorbed dextran gels, Figure S5: Elution of EB solution to Fe-DC, before purification (left), elution of dye (middle), after purification (right), Figure S6: Effect of adsorption capacity in the presence of humic acid (HA), Figure S7: (a) Amberlite^®^-410 resin-incorporated dextran gel column (left), Fe-DC (right), (b) After elution of EB solution (15 μM) to columns, Figure S8: Comparison of removal efficiency of EB dyes using adsorbent-incorporated columns, Table S1: Comparison of Fe_3_O_4_-based adsorbents in terms of their anionic dye removal efficiencies.
