# Supplementary material for: Continuous Flow Removal of Anionic Dyes in Water by Chitosan-Functionalized Iron Oxide Nanoparticles Incorporated in a Dextran Gel Column
Source: Nanomaterials (Basel). 2019 Aug 14;9(8):1164. doi: 10.3390/nano9081164 (PMC6724129; doi:10.3390/nano9081164)
Supplement: Supplementary file 1 [file nanomaterials-09-01164-s001.pdf]

Supporting Information

Continuous Flow Removal of Anionic Dyes in Water by Chitosan-Functionalized Iron Oxide Nanoparticles Incorporated in a Dextran Gel Column

Sang Yeob Lee [1], Ha Eun Shim [2], Jung Eun Yang [3], Yong Jun Choi [4] and Jongho Jeon [1,*]

[1] Department of Applied Chemistry, School of Applied Chemical Engineering, Kyungpook National University, Daegu 41566, Korea
[2] Department of Chemistry, Kyungpook National University, Daegu 41566, Korea
[3] Department of Advanced Process Technology and Fermentation, World Institute of Kimchi, Gwangju 61755, Korea
[4] School of Environmental Engineering, University of Seoul, Seoul 02504, Korea
* Correspondence: jeonj@knu.ac.kr

1. Hydrodynamic size and surface charge of chitosan-coated Fe_3O_4 nanoparticles

Chitosan-coated Fe_3O_4 nanoparticles were diluted 1/100 with distilled water and then 50 μL of sample was analyzed for size distribution and zeta potential by using a Zetasizer Nano-ZS90 (Malvern Instruments, UK).

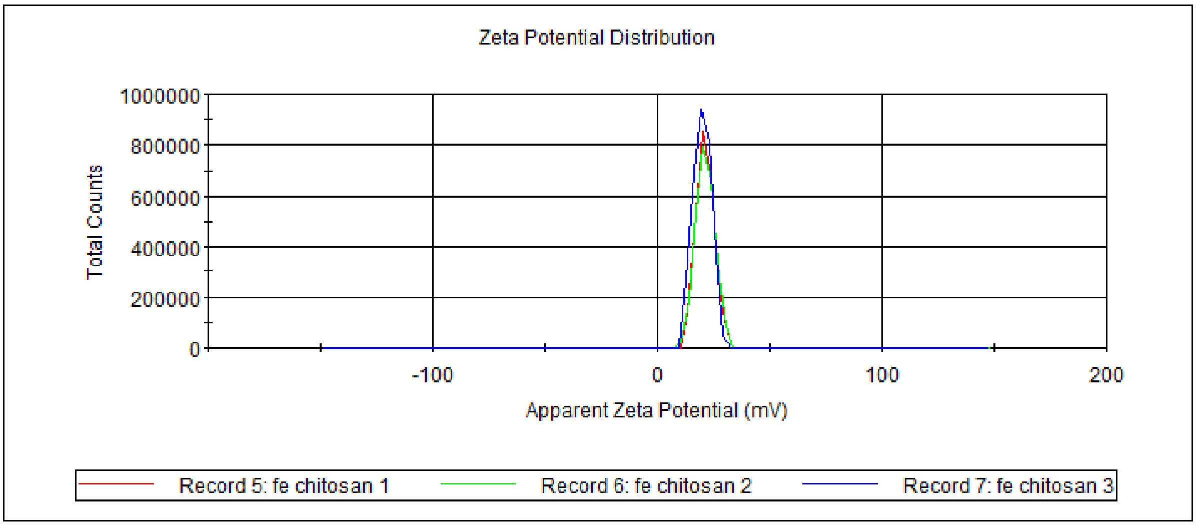

Zeta potential: 21.2 mV (± 3.91)

Figure S1. Zeta potential of the chitosan-coated Fe_3O_4 nanoparticles.

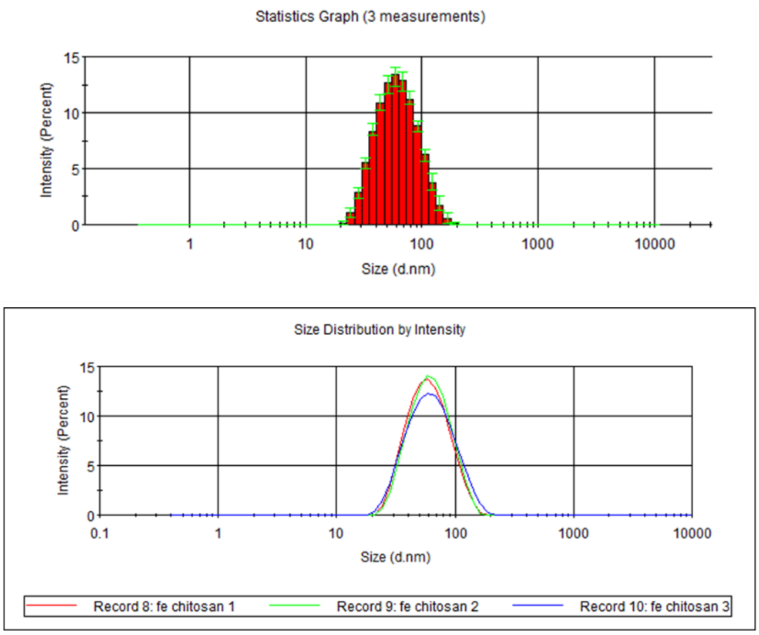

Size (nm)	Record 1	Record 2	Record 3
15.686	0	0	0
18.166	0	0	0
21.037	0	0	0.354
24.363	0.949	0.661	1.458
28.214	3.05	2.351	3.311
32.674	5.949	4.947	5.655
37.84	9.032	8	8.097
43.821	11.655	10.906	10.229
50.748	13.309	13.074	11.709
58.771	13.716	14.063	12.318
68.061	12.854	13.672	11.989
78.82	10.936	11.98	10.8
91.28	8.351	9.328	8.955
105.709	5.578	6.253	6.746
122.42	3.091	3.372	4.501
141.772	1.263	1.238	2.533
164.183	0.268	0.156	1.083
190.137	0	0	0.261
220.194	0	0	0
255.002	0	0	0

Hydrodynamic size: 65.24 ± 1.64 nm

Figure S2. Hydrodynamic size of the chitosan-coated Fe_3O_4 nanoparticles.

2. Photo images showing the preparation of Fe-DC

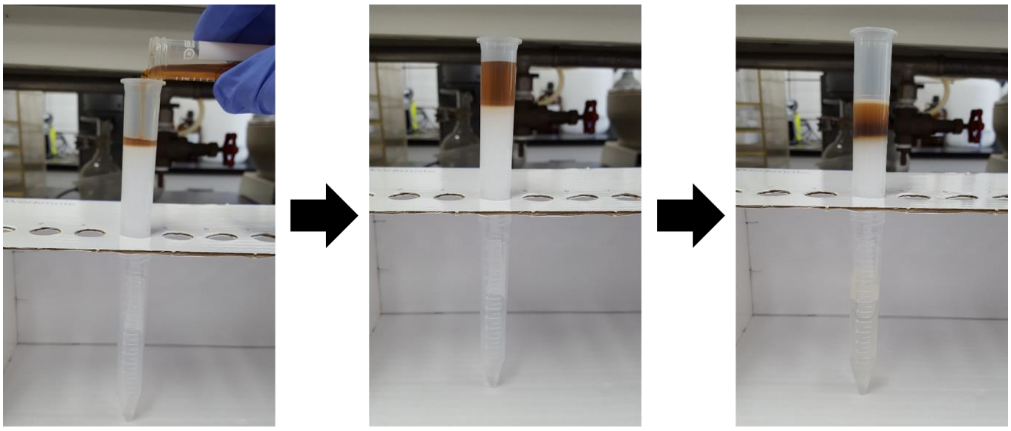

Figure S3. Incorporation of chitosan-coated Fe_3O_4 nanoparticles in the dextran gel column (PD-10).

3. SEM images of dye-adsorbed dextran gel beads (after purification step)

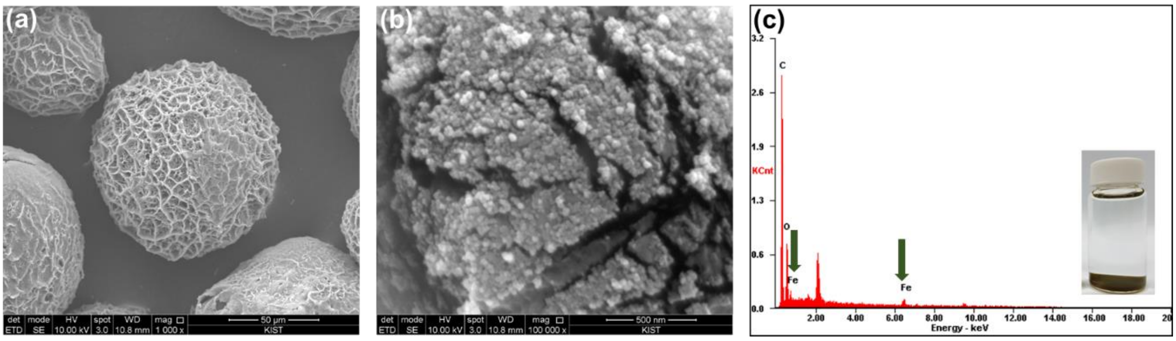

Figure S4. (a,b) SEM images of EB-adsorbed dextran gel beads, (c) EDX analysis of EB-adsorbed dextran gels. Arrows in figure (c) indicate the presence of iron elements on the surface the dextran gel beads.

4. Photo images showing the purification procedure using Fe-DC

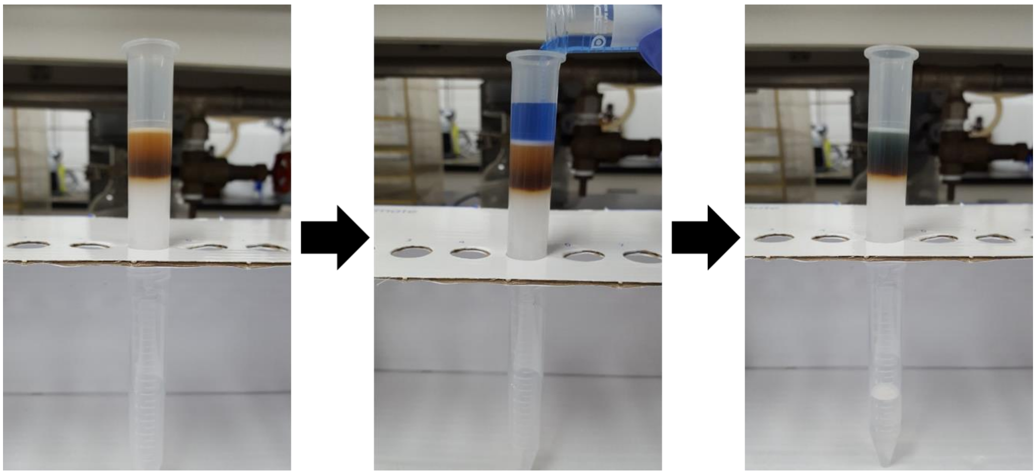

Figure S5. Elution of EB solution to Fe-DC, before purification (left), elution of dye (middle), after purification (right).

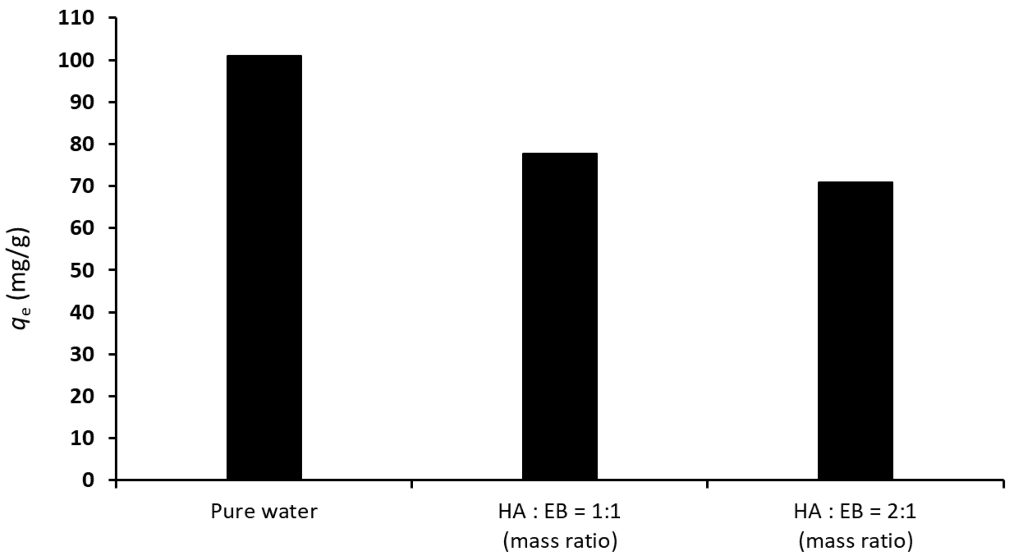

Figure S6. Effect of adsorption capacity in the presence of humic acid (HA). (Elution of 15 μM of EB, 10 mL).

Table S1. Comparison of Fe_3O_4-based adsorbents in terms of their anionic dye removal efficiencies.

Adsorbent	Anionic dye	Adsorbent used (mg)	C_0* (mg/L)	q_m** (mg/g)	Ref.
PVP coated Fe_3O_4	Reactive Orange 13	5	80	32.50	21
	Reactive Yellow 15			25.04	
$ZnFe_2O_4$	Evans Blue	50	50	45.45	34
$Ni_{0.5}Zn_{0.5}Fe_2O_4$	Methyl Blue	50	300	54.7	39
APTES coated Fe_3O_4	Sunset Yellow	10	10	91.74	36
DMDAAC coated Fe_3O_4	Methyl Blue	11	19	109.89	11
Lignin coated Fe_3O_4	Methyl Blue	20	50	211.42	20
PEI coated Fe_3O_4	Alizarin Red S	30	100	256.0	10
	Methyl Orange		100	242.4	
Fe-DC	Evans Blue	2.5	96.1	243.90	This work

* Initial dye concentration. ** Maximum adsorption capacity.

5. Comparison experiments with anion exchange resins

Different amounts (50 or 100 mg) of anion exchange resins (Amberlite® IRA-410 and Amberlite® IRA-900) were incorporated into the dextran gels desalting column. To evaluate removal efficiency, EB solution (15 μM, 10 mL) was added to adsorbent-incorporated columns. The removal efficiency (%) is defined by the Equation (4).

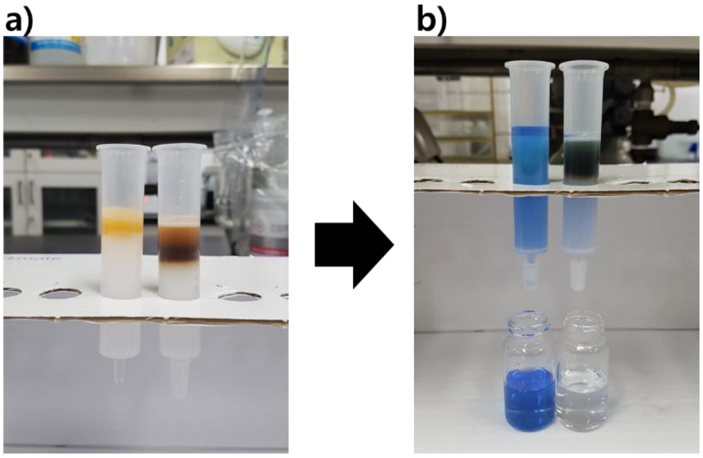

Figure S7. (**a**) Amberlite®-410 resin-incorporated dextran gel column (left), Fe-DC (right), (**b**) After elution of EB solution (15 μM) to columns.

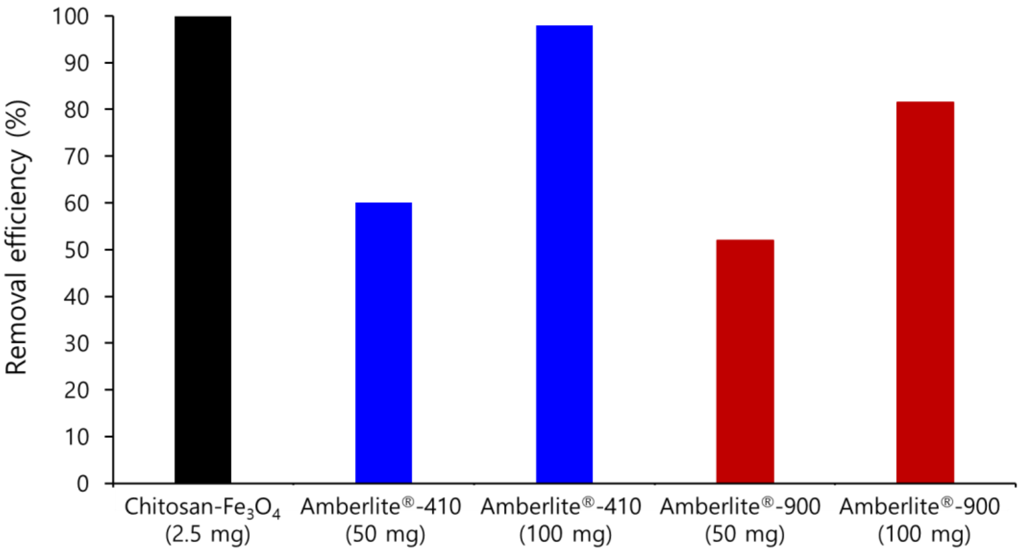

Figure S8. Comparison of removal efficiency of EB dyes using adsorbent-incorporated columns.
